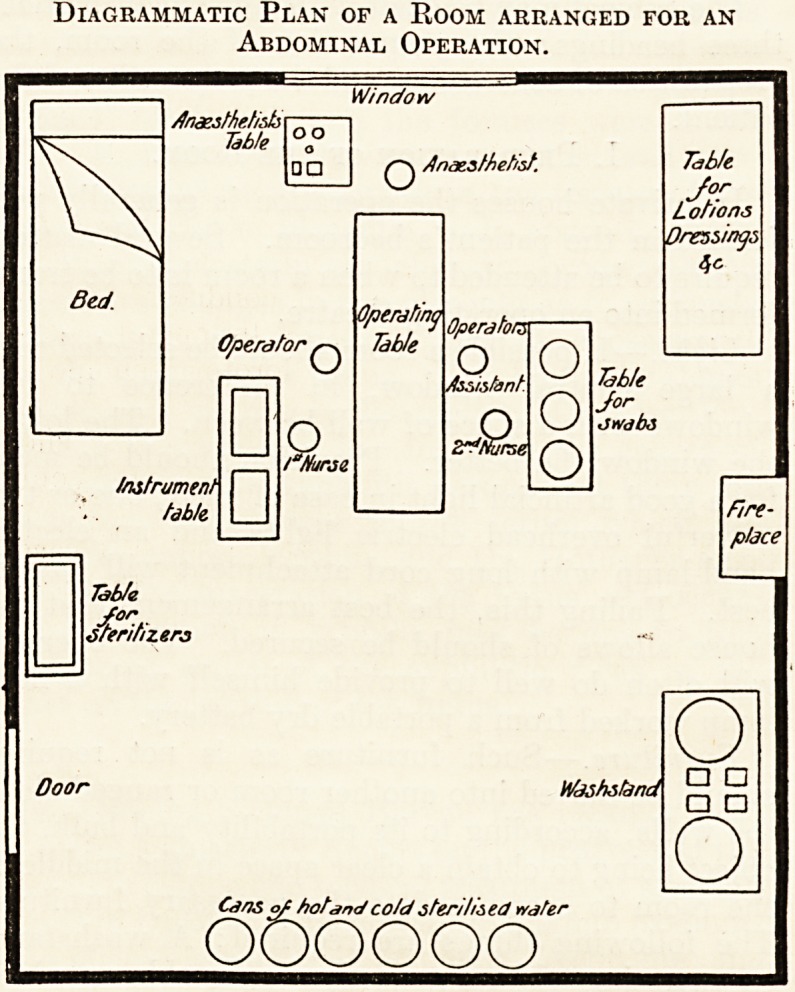# The Preparation and After-Treatment of Abdominal Operations

**Published:** 1908-12-19

**Authors:** Arthur E. Giles

**Affiliations:** Surgeon to the Chelsea Hospital for Women; Gynæcologist to the Prince of Wales's General Hospital, Tottenham


### Hospital Clinics.

### THE PREPARATION AND AFTER-TREATMENT OF ABDOMINAL OPERATIONS.

By ARTHUR E. GILES, M.D., B.Sc. Lond., F.R.C.S. Edin.; Surgeon to the Chelsea Hospital for Women; Gynæcologist to the Prince of Wales's General Hospital, Tottenham.

### I.—PREPARATION FOR ABDOMINAL OPERATIONS.

(A Post-Graduate Lecture delivered at the Prince of Wales's General Hospital, Tottenham, October 22, 1908.)

It has not infrequently happened that when an operation has been arranged for in a private house one has been asked by the medical attendant, "What do you like got ready?" The present observations are intended to serve as an answer to this question. I have supposed the case of an abdominal operation, because what applies to this applies, with very few modifications, to all gynæcological operations, and indeed to operations in general.

The subject may be dealt with conveniently under three headings: the preparation of the room, the preparation of accessories, and the preparation of the patient.

#### I. Preparation of the Room.

In private houses the operation is generally performed in the patient's bedroom. Several matters require to be attended to when a room is to be transformed into an operating theatre.

*Light.*—If possible a room should be selected with a large central window, in preference to two windows with a space of wall between. The loftier the window the better. Provision should be made for a good artificial light in case of need; one or two powerful overhead electric lights and an electric hand-lamp with long cord attachment will answer best. Failing this, the best arrangement that the house allows of should be secured. The operator will often do well to provide himself with a head lamp worked from a portable dry battery.

*Furniture.*—Such furniture as is not required should be moved into another room or ranged along the walls, according to its portability and bulk, the object being to obtain a clear space in the middle of the room to accommodate the necessary furniture. The following things are required: A washstand, an operating-table, a good-sized side table on which to place bowls, dressings, and other things, three small tables to accommodate respectively the instrument trays, the bowls for the swabs, and the anæsthetist's requisites; a chair or stool for the anæsthetist, and the patient's bed. The tables should be firm and of a convenient size; they should be covered with clean towels, and if they have polished tops, a piece of mackintosh or several layers of paper should be spread over them under the towels. We shall consider the arrangement of the furniture after dealing with the accessories required.

*Floor and Curtains.*—It is a mistake to suppose that it is always necessary to take up the carpet. If the floor is covered all over with linoleum, loose rugs and carpets can be removed and the linoleum washed. This is the best arrangement. But in the case of a fitted carpet, the best plan is to lay down large pieces of brown paper or newspaper and then spread a large drugget, which can be stretched and lightly tacked down. A dust-sheet answers the same purpose. Failing these, paper alone can be used to protect the carpet. Heavy curtains should be removed if they obstruct the light, otherwise they are best left alone. A short muslin curtain can be fastened up over the lower half of the window, leaving the upper half clear.

*Cleaning the room.*—Excess of zeal is to be deprecated. Woodwork, and especially ledges of all kinds, can be wiped over with a damp cloth, but an active dusting should be avoided, especially on the day of the operation. Ledges covered with thick dust and clear air in the room are vastly to be preferred to clean ledges and a dust-laden atmosphere. It is usually advisable to "let sleeping germs lie," rather than drive them out of their accustomed haunts to float about till perchance they come to rest in the patient's abdomen. Naturally, when an operation is arranged several days ahead it is best to have a room thoroughly cleaned out; but it must be done in such a way that the atmosphere of the room has time to clear. On the night before the operation it is advisable that the patient should sleep in another room, so that preparations can be carried out in the operating room in the morning without disturbing or alarming her.

#### II.—Preparation of Accessories.

We have enumerated the articles of furniture required, and we must now consider the other stage properties required for the proper presentation of the operative drama.

*Hot and Cold Water.*—This is required in abundance, and it must be sterilised. A quantity of water should be boiled the night before and stored in clean cans or jugs, whose mouths are then tied over with gauze. In this way we shall have plenty of cold sterilised water when we want it. On the morning of the operation more water is boiled and stored ready for use, and two or three kettles of boiling water should be available for use in the sterilisers.

*Towels.*—A couple of dozen of these will not be too many. They are required, not only for the drying of hands, but for covering over the patient, for the anæsthetist's use, and for drying the patient and the instruments at the end of the operation. It is very convenient to have half a dozen dry towels that have been sterilised in a drum in a Schimmelbusch or other steriliser, and when such a steriliser is available it can be used also for swabs, dressings, and overalls. Otherwise half a dozen towels can be boiled in a clean saucepan and wrung out as required.

*Mackintoshes.*—Four of these are useful, two fairly large ones to go on the patient's bed and under the patient on the operation table, and two smaller ones to be placed, covered with sterilised towels, above and below the field of operation. Jaconet answers quite well for the two smaller mackintoshes.

*Hot-water Bottles.*—It is advisable to have three or four of these, preferably the india-rubber ones. Stone bottles will do, however, and in an emergency wine or beer bottles can be filled with hot water, or a brick can be heated in the oven and wrapped round in flannel. One hot-water bottle is placed between the patient's feet during the operation, and two or three are put into the bed before the patient's return. Many operators make it a rule never to allow a hot-water bottle in bed with an unconscious patient.

*Bowls and Dishes.*—The following should be provided if possible: Three flat enamelled iron or porcelain dishes for instruments, six good-sized enamelled bowls for swabs, towels, gloves, and lotions, six smaller bowls for various purposes, such as receiving parts removed, catching discharges, holding small quantities of lotion, or for the anæsthetist's use if the patient is sick. A large footbath should be placed under the table to take soiled things, and two or three kidney-trays come in useful.

*Washing Materials.*—Two new nail-brushes should be boiled and placed in a bowl of lysol ready for the use of the operator and his assistant; two washing-basins and two pieces of soap should also be provided.

*Lotions.*—Different operators have different tastes in the matter of lotions. They are used almost exclusively for the hands, since everything else that comes in contact with the wound can be sterilised by heat. Lysol of the strength of 1 drachm to the pint is always useful. My own preference is for biniodide of mercury—namely, one bowl of 1 in 1,000 in rectified or methylated spirit, and one bowl of 1 in 2,000 in water. The hands are soaked first in the spirituous and then in the watery solution.

*Gloves.*—I may say a word here as to the use of rubber gloves, although the provision of these usually devolves upon the operator. They have been found to be a remarkable safeguard against infection, but they in no way lessen the necessity for careful preparation of the hands. Their value is threefold. In a clean case they protect the patient, in a septic case they protect the operator, and when the surgeon has had to deal with a septic case he can proceed to operate on a clean case with an easy mind. It is obvious that they present enormous advantages in connection with midwifery practice. To prepare gloves it is only necessary to boil them for five or ten minutes, and they are then placed in sterilised water or weak biniodide, and well filled with liquid before the hands are introduced.

*Swabs and Dressings.*—For an ordinary abdominal operation one dozen swabs are sufficient. They are made of six or eight folds of gauze, sewn round the edges, or of flat square pads of gamgee tissue sewn into gauze. Four should be large, about eight inches square or six by eight, and eight should be small, about four inches square. It is useful to have a reserve of twelve more small swabs, tied up in two packets of six in each, to be used only in case of need. They are sterilised in a Schimmelbusch, or by boiling in a clean saucepan. At the time of the operation the twelve swabs are placed in a large bowl of sterilised water, and wrung out as required. After use they are placed in a second bowl of sterilised water in which they can be rinsed out, and transferred to a third bowl ready for use. The dressings consist of plain sterilised gauze and absorbent wool, which are either sterilised just before use and kept in a drum till required; or bought in packets ready sterilised. A many-tailed flannelette binder is prepared by the nurse, being made of a size to fit the patient.

*Overalls.*—These are generally brought by the surgeon, but they can also be obtained ready sterilised in drums. There should be sufficient to supply the operator, his assistant, the anæsthetist, and the two nurses.

*Arrangement of the Operating Room.*—A convenient arrangement is that shown in the diagrammatic plan herewith.

III.—Preparation of the Patient.

In order to obtain the best results in abdominal surgery there are various matters that should be carefully inquired into beforehand, because there are certain conditions that interfere with the smoothness the convalescence, and others that so increase the danger of operation that they can only be disregarded in urgent cases where life is immediately threatened.

Diagrammatic Plan of a Room arranged for an Abdominal Operation.

*Preliminary Examination of the Patient.*—The heart, respiratory system, and urine should always be carefully examined. Many patients inquire anxiously whether their heart is strong enough to stand an operation. As a matter of fact there are very few cases in which there is sufficiently serious heart disease to cause any anxiety, and even grave heart disease is not a bar in an urgent case. The patient must then run the risk. Valvular disease seldom gives trouble. The most dangerous cases are those of fatty heart and inadequate compensation. But in the presence of any form of heart disease special care is required, particularly when the time comes for the patient to get up. When fatalities have occurred they have taken place much more often after the patient has got up than as the immediate result of the operation.

The presence of varicose veins, and especially of phlebitis, must prepare us to look out for thrombosis and embolism during convalescence, and entails extra care in allowing the patient to get up.

Bronchitis is often a source of anxiety. In the acuter stages an operation should never be performed unless very urgent; but when it is of the chronic form operation may be undertaken, with the proviso that ether should be avoided for the anæsthesia, and that every care should be taken to get the operation through as quickly as possible. When adenoids are present they may complicate the anæsthesia, and when time allows it is much better to have them removed before undertaking an abdominal operation. Diabetes is a contra-indication to operations of expediency; but the presence of glycosuria apart from diabetes is no bar; in some cases it clears up rapidly after operation.

Nephritis is another formidable complication, and when casts are found in the urine in addition to albumin an operation should always be deferred, except in urgent cases, until the renal condition has improved. When it is necessary to operate on a patient who has albuminuria, chloroform should be preferred to ether as an anæsthetic. The same remarks apply to cases of chronic alcoholism: such patients are bad subjects for operation, and only great necessity should induce us to operate on them.

Cases of mental instability and borderland cases should be undertaken with great reserve; these are the patients who are liable to develop acute mania and melancholia after operations, and they have supplied the bulk of the examples that have led to the tradition that operation on the pelvic organs causes insanity. I am persuaded that neither ovariotomy nor hysterectomy is followed by insanity except in the case of those with a marked predisposition. It must also be borne in mind that many women have become neurasthenic and mentally unstable as the result of their pelvic disorders; and in such patients the operation is often followed by definite improvement in the mental condition.

*Preliminary Treatment.*—It is a great advantage that a patient should be kept in bed for two or three days before an abdominal operation; the rest quietens the nervous system, diminishes post-operative shock, and allows of proper attention to the bowels. Aperients should be given two or three times if necessary, followed each time by an enema. Some patients have loading of the colon of long standing, and the results of successive enemas will amaze any one who has not had experience in these matters. The unloading of the colon not only minimises trouble after the operation, but also promotes flaccidity of the bowels at the time, and thus materially facilitates the performance of the operation. Another good result of attention to the bowels is the lessening of post-operative sickness. Some operators administer small doses of strychnine at intervals beforehand; I have not generally found this necessary, but in some cases with marked anæmia and a weak heart it has a good effect. The mouth should be carefully attended to in order to eliminate oral sepsis and its possible result, parotitis. For the rest it is important that the patient should be cheered up and encouraged.

*Washing.*—On the evening before operation the patient should have a warm bath if her condition allows; otherwise she should be thoroughly washed in bed.

*Douching* is not absolutely necessary as a matter of routine, but it is always salutary and often necessary. Strong antiseptics should not be used; something mild is sufficient, such as biniodide of mercury (1 in 6,000), sanitas, weak lysol, or boracic acid. The douche should be given twice a day up to and including the morning of the operation day.

*Preparation of the Abdomen.*—The pubes and vulva should be shaved, and the abdomen, vulva, and adjacent parts of the thigh well washed with soap and water—hard scrubbing does more harm than good. The soap is washed off and the skin is then well cleansed with ether or terebene; this is washed off with spirit and a final cleansing is made with 1 in 1,000 biniodide of mercury. This process can be carried out when the patient is under the anæsthetic; and with a very nervous individual this course is advisable. It is often more convenient, however, to prepare the abdomen beforehand, namely, on the previous evening, when an operation is to take place early in the morning; or in the morning when it is arranged for the afternoon. In either case it is necessary, after the preparation, to apply a compress of lint wrung out of a weak biniodide solution (1 in 6,000) with a piece of jaconet to keep it moist and a many-tailed bandage to hold it all in place. In the case of any operation that will affect the integrity of the vaginal vault, especially hysterectomy, it is advisable to pack the vagina with sterilised or double-cyanide gauze after the evening douche, and again after the morning douche.

*Food.*—The last meal should be taken at least five hours before the operation, and it should be a light one. When the operation is timed for the early morning a light dinner or supper should be taken the night before, and a cup of beef tea in the small hours of the morning. For an afternoon operation the patient should have a light breakfast and nothing afterwards.

*Clothing.*—A nightdress opening down the back should be worn for the operation, and over this a dressing-jacket or gown put on back to front, so that it can be removed easily when the patient is on the table. The chest should be warmly covered; the best arrangement is a kind of jacket of cotton wool under the nightdress. The legs should be clad in clean long woollen stockings reaching up to the top of the thighs; or if these cannot be obtained a pair of stockings and pyjama-trousers will do very well as a make-shift.

*Catheter.*—When there is any doubt whether the bladder has been completely emptied the catheter should be passed before the operation. It is also advisable in the case of tumours filling up the pelvis, as the bladder is then liable to be displaced. In ordinary cases the passing of the catheter need not be done as a matter of routine: it is sufficient if the patient is allowed to pass water naturally before going on to the operation table.

## Figures and Tables

**Figure f1:**